# Ventilator-Induced Lung Injury: The Unseen Challenge in Acute Respiratory Distress Syndrome Management

**DOI:** 10.3390/jcm14113910

**Published:** 2025-06-02

**Authors:** Raffaele Merola, Maria Vargas, Denise Battaglini

**Affiliations:** 1 Anesthesia and Intensive Care Medicine, Department of Neurosciences, Reproductive and Odontostomatological Sciences, University of Naples Federico II, 80131 Naples, Italy; rafmerola2003@gmail.com (R.M.); vargas.maria82@gmail.com (M.V.); 2 Department of Surgical Sciences and Integrated Diagnostics (DISC), University of Genoa, 16132 Genova, Italy; 3 Anesthesia and Intensive Care, IRCCS Ospedale Policlinico San Martino, 16132 Genova, Italy

**Keywords:** mechanical ventilation, ventilation-induced lung injury, acute respiratory distress syndrome, ARDS, VILI

## Abstract

Invasive mechanical ventilation is a cornerstone therapy for supporting patients with acute respiratory distress syndrome (ARDS) by relieving respiratory muscle strain and ensuring gas exchange. Despite its life-saving benefits, mechanical ventilation can induce ventilator-induced lung injury (VILI), a critical condition characterized by mechanisms such as barotrauma, volutrauma, atelectrauma, ergotrauma, and biotrauma. This review examines the pathophysiological mechanisms of VILI and their impact on lung function, particularly in patients with ARDS. It highlights the importance of lung-protective ventilation strategies, including low tidal volume and tailored positive end-expiratory pressure, which have been shown to improve outcomes in ARDS. The role of prone positioning in enhancing lung homogeneity and improving outcomes is also discussed. Furthermore, emerging concepts such as mechanical power and individual respiratory mechanics are explored as potential avenues for personalized ventilation strategies. Despite advancements, the optimal approach to mechanical ventilation remains a subject of ongoing research.

## 1. Introduction

The primary objective of invasive mechanical ventilation (MV) is to reduce the workload of the respiratory muscles while maintaining adequate gas exchange. MV played a crucial role during the polio health crisis, leading to a notable decrease in mortality rates [1,2]. However, despite its clear therapeutic advantages, MV is not without risk, as it can inflict damage on the lungs, potentially resulting in what is known as ventilator-induced lung injury (VILI). The negative impact of mechanical MV dates to 1744, when John Fothergill reported the case of a successfully resuscitated patient from coal fume exposure using mouth-to-mouth ventilation. This suggested that this technique might outperform MV, as human lungs can tolerate an external respiratory force [3]. Indeed, the lungs of MV patients can develop alveolar infiltrates and hyaline membranes [4]. More recently, attention has shifted to the worsening damage MV can cause in already compromised lungs, as well as the harm it can initiate in healthy lungs. However, the clinical relevance of VILI was definitively proven only 250 years later by a study that showed that a ventilatory strategy aimed at minimizing such damage led to a significant reduction in mortality among patients with acute respiratory distress syndrome (ARDS) [5].

Considering this, this review aims to explore the mechanisms underlying this condition, its impact on the pathophysiology of the patients with ARDS, and the clinical approaches to prevent and reduce its impact on outcomes.

## 2. Pathophysiological Mechanisms

Initially, four mechanisms of VILI were identified: barotrauma, volutrauma, atelectrauma, and biotrauma. More recently, ergotrauma and heterogeneous respiratory mechanics have been identified as contributing factors to VILI, leading research toward the personalization of lung-protective MV. Additional factors—such as respiratory acidosis, respiratory rate, pulmonary vascular pressures, and body temperature—have been experimentally linked to the onset of VILI.

### 2.1. Barotrauma and Volutrauma

Preliminary evidence of barotrauma and volutrauma comes from the experimental setting. Initially, barotrauma lung injury mediated by high inflation pressure, and volutrauma lung injury mediated by overdistension, were considered distinct, yet related, entities.

In 1974, Webb and Tiemey, first demonstrated that rats MV at high positive pressures developed lung injury that was proportional in severity to the pressures used and characterized by functional and structural changes. In this study, rats were subjected to MV with very high peak airway pressures, resulting in lung overdistension and hypoxemia, as postmortem analysis revealed both perivascular and alveolar edema. Conversely, rats ventilated at the same pressures but with an added 10 cm H_2_O of positive end-expiratory pressure (PEEP) did not exhibit edema, emphasizing the significance of end-expiratory lung volume (EELV) in achieving protective MV. The precise mechanisms underlying this protective effect remain incompletely understood [6]. Dreyfuss et al. demonstrated that MV with large tidal volumes (V_T_) can increase lung alveolar permeability and trigger inflammatory responses, resulting in pulmonary edema in animals. This pointed to volume-induced lung stretch as the primary cause of injury, rather than airway pressure alone, leading to the concept of “volutrauma” [7].

A few years later, the concept of “baby lung”, also known as the residual aerated volume, was introduced. The baby lung is directly linked to the damage induced by V_T_, which is intimately linked to EELV, a factor that determines dynamic strain, which is intrinsically associated with higher lung inflammation. While the concept of the “baby lung” has evolved over time, the fundamental principle has stayed the same: a normal V_T_ administered to small baby lungs in ARDS patients may result in substantial supraphysiological tissue stresses and injury. Quantitative computed tomography (CT) scans allowed an understanding of the relationship between the size of the open lung and respiratory system compliance (Crs)—a measurement of the mechanical properties of the lung and chest wall. The lung may not be inherently stiff but rather may have a decreased volume. These results cast doubt on long-held beliefs about lung rigidity and emphasize how crucial lung size is to understand respiratory function and VILI [8,9,10,11].

**Figure 1:**
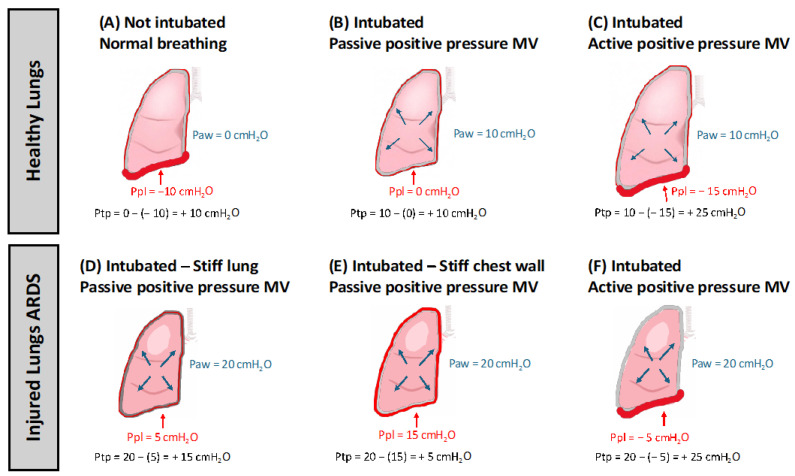
Mechanisms of Barotrauma and Volutrauma: The Role of Transpulmonary Pressure. This figure illustrates how transpulmonary pressure (Ptp)—the difference between alveolar pressure (Palv) and pleural pressure (Ppl)—determines the risk of alveolar overdistension in different clinical and physiological scenarios. (**A**) illustrates end-inspiration in a spontaneously breathing individual with healthy lungs. At this point, Palv remains at 0 cm H_2_O, while Ppl is negative (−10 cm H_2_O), resulting in a transpulmonary pressure (Ptp) of +10 cm H_2_O. (**B**) depicts the same spontaneously breathing individual with healthy lungs during passive positive-pressure ventilation, using the same tidal volume as in Panel A. Despite the change in ventilation mode, the degree of lung inflation is comparable, with a Palv of +10 cm H_2_O and a Ppl of 0 cm H_2_O, resulting in a Ptp of +10 cm H_2_O. (**C**) depicts the same healthy lung under active positive-pressure ventilation. Palv reaches +10 cm H_2_O, while the marked negative Ppl (−15 cm H_2_O) generated by the patient’s effort results in a Ptp of +25 cm H_2_O. (**D**) shows an intubated patient with acute respiratory distress syndrome (ARDS) and stiff lungs under passive positive-pressure MV. Despite a high Palv of +20 cm H_2_O, the Ppl rises to +15 cm H_2_O due to reduced lung compliance, resulting in a Ptp of only +15 cm H_2_O. (**E**) shows an intubated ARDS patient with a stiff chest wall (such as one with a pleural effusion, massive ascites, or severe obesity). In these patients, a significant portion of the ventilator-delivered pressure is used to inflate the chest wall rather than the lungs. As a result, the plateau pressure may be high, but so will the pleural pressure, maintaining the transpulmonary pressure at values that avoid the risk of alveolar overdistension. (**F**) shows an ARDS patient with marked dyspnea on mechanically assisted ventilation (e.g., pressure support ventilation). In such cases, a marked inspiratory effort causes a large negative pleural pressure swing, increasing transpulmonary pressure to potentially injurious levels, even if the airway pressure remains low (e.g., 20 cmH_2_O). These examples highlight the importance of assessing transpulmonary pressure—not just airway pressure—when evaluating the risk of ventilator-induced lung injury (VILI).

Similar results emerged in other experimental studies, leading to the misleading conclusion that volutrauma and barotrauma were two distinct entities, and that the former had more clinical importance than the latter [12,13,14,15]. Further evidence that high airway pressures are not necessarily harmful came from the observation that trumpet players, despite generating airway pressures of approximately 150 cmH_2_O, much higher than those found in the airways of MV patients, did not develop any damage to the lung parenchyma [16]. In fact, high inspiratory airway pressure is not to be considered a reliable index of alveolar distension. According to the equation of motion of the respiratory system, in fact, each breath requires pressure to inflate the lungs, which includes overcoming both airway resistance and inertia the pressure gradient required to accelerate the gas, as well as overcoming the elastic properties of the respiratory system. Furthermore, even airway pressure measured in the absence of flow, such as plateau pressure (P_PLAT_), obtained with a temporary occlusion of the flow at the end of inspiration, and therefore proportional to the elastance of the respiratory system, is not to be considered accurate for measuring alveolar distension. P_PLAT_ is, in fact, spent partly on alveolar distension and partly on chest wall distension. The pressure spent on alveolar distension is the transpulmonary pressure, defined as the difference between alveolar pressure and pleural pressure. Consequently, lung volume and transpulmonary pressure are closely related [17]. It can therefore be stated that one of the mechanisms that causes VILI is excessive transpulmonary pressure that determines the pathological distension of the ventilated alveolar units and that, as a consequence of this, the distinction between barotrauma and volutrauma becomes purely theoretical [18]. Therefore, not considering transpulmonary pressure as an indicator of alveolar distension can lead to overestimating or underestimating the risk of VILI. Pressures delivered by the ventilator are often used to expand a stiff chest wall rather than the lungs. In this case, high airway pressure, specifically plateau pressure, might not be a reliable indicator of excessive pulmonary distending forces. When pleural pressure rises alongside airway pressures, pneumothorax and lung overdistension are less likely to occur. However, in patients with ARDS with heterogeneous lungs, even modest airway pressures may produce very high pleural pressures, resulting in a substantial lung stretch and elevated transpulmonary pressures (Figure 1) [19].

### 2.2. Atelectrauma

In the context of ARDS, surfactant dysfunction, increased weight of edematous lung tissue as well as supine position contribute significantly to the development of regional atelectasis [20]. The cyclical process of opening and collapsing of these atelectatic lung units, which are still recruitable, during tidal ventilation, is a key factor in the development of lung injury. “Atelectrauma” is characterized by epithelial shedding, hyaline membranes, and pulmonary edema [21]. For atelectatic alveoli, a significant amount of shear stress is produced during recruitment, arising at the junction between the inflating air and the collapsed airway [22]. This shear force leads to mechanical damage [23]. The mechanical strain on the lung parenchyma at the intersections of the aerated and atelectatic regions may be four to five times higher than that seen in other lung regions [24].

Recent insights into VILI have shifted attention beyond the traditional “baby lung” paradigm, emphasizing the role of microenvironmental factors in the pathogenesis of injury. In ARDS, the lung exhibits marked heterogeneity in inflation, resulting in a non-uniform distribution of stress and strain. This disparity is accentuated in regions with impaired surfactant function, where repetitive alveolar recruitment and derecruitment generate atelectrauma and give rise to “stress multipliers”—areas in which collapsed, or fluid-filled alveoli transmit excess mechanical forces to adjacent, compliant units. Consequently, overdistension may occur not within the residual functional lung, but in neighboring healthy tissue via alveolar interdependence, thereby contributing to volutrauma [25,26,27,28]. These focal injuries, often referred to as “hidden micro-atelectasis”, typically elude detection by conventional imaging modalities at the bedside [25]. Animal studies corroborate this evolving concept, demonstrating that static high airway pressures—when coupled with adequate PEEP—may be well tolerated, whereas dynamic strain from insufficient PEEP can provoke substantial injury [29,30]. These findings call for a reexamination of ventilatory strategies, advocating for approaches that limit not only global overdistension but also regional stress propagation, with the goal of mitigating progressive VILI.

From a clinical perspective, the use of low-V_T_ ventilation may play a crucial role in minimizing atelectrauma by maintaining low airway driving pressures. This approach reduces the likelihood of surpassing the critical opening pressure that would otherwise cause the collapsed lung units to be overstretched [31]. In addition, the application of PEEP set above the critical closing pressure of lung units that are prone to collapse promotes their sustained recruitment, potentially offering additional protection against atelectrauma [32,33,34].

However, it is important to underline that the optimal strategy for titrating PEEP remains to be fully determined. Indeed, although PEEP can improve oxygenation, ventilation, and lung compliance by recruiting dense or atelectatic areas, in patients with ARDS and focal distribution of lung consolidations it can cause alveolar overdistension and increased inflammation [35,36].

### 2.3. Biotrauma

**Figure 2:**
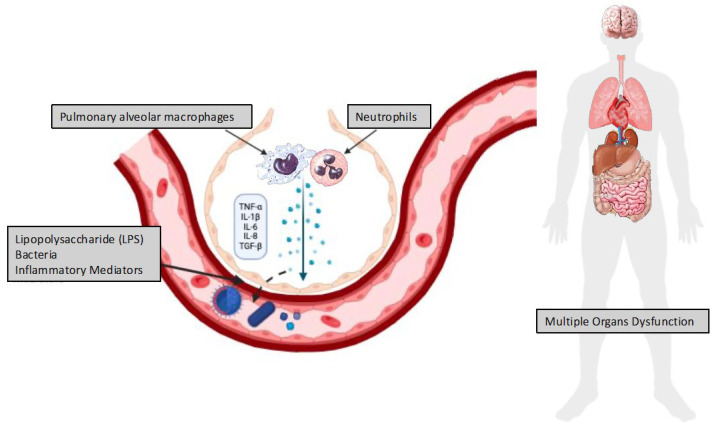
Biotrauma: The Inflammatory Cascade Triggered by Mechanical Lung Injury. This figure illustrates the pathophysiological mechanisms underlying biotrauma, a form of ventilator-induced lung injury (VILI) resulting from the mechanical stress of ventilation. Mechanical forces damage alveolar and airway structures, leading to the release of intracellular mediators from epithelial, endothelial, and immune cells. These mediators exert multiple effects: some directly injure lung tissue, while others activate intracellular signaling pathways that amplify inflammation and promote fibrotic remodeling. Key inflammatory mediators—such as tumor necrosis factor (TNF-a), interleukins (IL), and transforming growth factor (TGF)—serve as homing signals that recruit immune cells (e.g., neutrophils and macrophages) to the lungs. Once activated, these cells release additional proinflammatory and cytotoxic molecules, perpetuating tissue damage and inflammation. In acute respiratory distress syndrome (ARDS), increased alveolar–capillary permeability allows the translocation of harmful substances, including cytokines, bacteria, and lipopolysaccharides, into the bloodstream. This systemic spread contributes to multiorgan dysfunction and may ultimately result in organ failure and death. The figure emphasizes how localized mechanical injury can evolve into a self-sustaining and system-wide inflammatory process with life-threatening consequences.

Mechanical lung injuries have the potential to either directly (through cell injury) or indirectly (through the activation of signaling pathways within epithelial, endothelial, or inflammatory cells) result in the release of different intracellular mediators [37,38,39]. While certain mediators may contribute to the formation of pulmonary fibrosis later, others may directly cause cytotoxicity to the lung tissue. Furthermore, some mediators can serve as chemoattractants, drawing immune cells, including neutrophils, to the lungs where they can expel more harmful chemicals. The term “biotrauma” refers to this process, where bacteria, lipopolysaccharides, or inflammatory mediators may translocate from the respiratory system into the systemic circulation by altered alveolar–capillary permeability, which is typical of ARDS. This may then contribute to multiorgan dysfunction and, ultimately, death (Figure 2) [37,40,41,42,43]. This concept has been confirmed through clinical trials, which have demonstrated that lung-protective ventilation strategies are effective in reducing systemic inflammation and in mitigating the failure of extrapulmonary organ systems, such as the cardiovascular, renal, and hepatic systems in patients with ARDS [37,40,43,44].

### 2.4. Ergotrauma and Respiratory Mechanics

The energy provided to the lungs per unit of time during MV is known as mechanical power (MP), and it has lately become a novel variable to account for lowering the incidence of VILI and improving the outcomes for ARDS patients [45]. As pressure is used to transport the V_T_ into the lungs during MV, electrical energy is transformed into potential, kinetic, and thermal energy. This energy transfer may put the lung parenchyma under mechanical stress, changing its structure at the cellular and tissue levels and contributing to lung damage [46,47,48]. MP, expressed in Joules per minute (J/min), offers a comprehensive assessment by integrating factors such as tidal volume, respiratory rate (RR), and inspiratory pressure into a single metric. This holistic measure allows clinicians to evaluate the total energy delivered to the lungs during each breath, which is crucial in understanding the cumulative effects of ventilation over time. To apply this concept at the bedside multiple mathematical formulas have been proposed [45,49,50].

The often-mentioned safety criteria of 17 J/min for MP may not even be relevant to all patients, and studies on MP have mostly examined patients with ARDS. Even though several research have included patients without ARDS, there is still no proof that MV techniques that target low MP are beneficial in this population. In critically ill patients, higher MP has been associated with increased mortality, with rates over 17 J/min in general critically ill patients with ARDS and over 22 J/min in those with ARDS [51,52]. Experimental evidence suggested that lung damage was even more severe and possibly fatal when MP values were higher than 25 J/min [53]. In patients receiving extracorporeal membrane oxygenation (ECMO) MP over approximately 14 J/min during the first three days was associated with increased mortality [54]. Despite these findings, the therapeutic use of MP is still unknown, mostly because of difficulties in accurately measuring and interpreting it. On the other hand, other parameters, such as driving pressure (ΔP), have been validated as mortality predictors and are simpler to measure at the bedside [55]. Costa and coworkers developed a new formula: (4 × ∆P) + RR [56]. This formula, as well as ∆P and RR, showed a significant association with mortality and poor neurologic outcome at six months in a cohort of post-cardiac arrest patients [57].

Lung tissue exhibits viscoelastic properties, indicating that stress varies over time during sustained strain rather than remaining constant. For instance, when the lungs are inflated at a constant volume, the transpulmonary pressure gradually decreases over time. Tissue deformation is represented as strain, defined as the ratio of V_T_ to the EELV. Strain serves as a metric to establish safe V_T_ thresholds aimed at preventing VILI. Additionally, the “strain rate” refers to the rate of change in lung strain (deformation) over time. Longer times are associated with lower strain rates, while shorter times correspond to higher strain rates.

Lung tissue has viscoelastic characteristics, meaning that the stress it experiences changes over time when subjected to a constant strain rather than remaining uniform. For example, when the lungs are held at a fixed volume, the transpulmonary pressure gradually declines. Strain, which is the deformation of the tissue, is quantified as the ratio of V_T_ to EELV. This strain measurement is crucial for determining safe V_T_ thresholds to help avoid VILI [58]. Moreover, the “strain rate” describes how quickly lung strain changes over time. When the duration of strain is extended, the strain rate tends to be lower, while shorter durations lead to higher strain rates. Essentially, this means that the speed at which the lung tissue deforms can significantly impact its health and risk of injury [58].

This concept is relevant to all aspects of mechanical ventilation. One important but often overlooked factor is the timing of changes in ventilator settings [58]. Ventilatory settings do not always account for the varying time constants during both inspiration and expiration, nor the uneven distribution of ventilation across different alveolar units. Even in healthy lungs, alveolar inflation and deflation do not occur at the same time. A low inspiratory time constant causes alveoli to fill up more rapidly during inhalation, while a high inspiratory time constant causes alveoli to take longer to fully inflate. This indicates that while certain alveoli can inflate quickly, others take longer to reach their full potential. Since not every portion of the lung is functioning at the same rate during ventilation, and especially in ARDS, this asynchronous activity may have an impact on gas exchange and total lung function. This means that at a high RR, alveoli will have less time to inflate, even if the time constant is low. RR is often overlooked, but recent research has linked it to VILI and mortality [56]. Lung injury results from the so-called stress relaxation that may depend on how quickly V_T_ or strain changes over time. V_T_ changes are often made suddenly, but the extracellular matrix needs time to relax the stress and reduce strain. Studies have shown that a shorter adaptation time (instead of abrupt changes) helps minimize lung injury. However, if the adaptation period is prolonged, more lung damage occurs, indicating that an injurious strain is inevitable. By employing a shorter adaptation period, it can be decreased [59].

Recruitment maneuvers (RMs), associated with improvements in oxygenation and lung mechanics, have been recognized as potential contributors to VILI. For RMs to be effective, the applied pressure must exceed the critical opening pressure of the small airways, and alveoli in heterogeneous lungs recruit at different time constants, meaning each alveolar unit requires a different timing to open. This emphasizes the importance of whether increases are rapid or gradual, as abrupt fluctuations in airway pressure and flow can elevate mechanical stress and potentially aggravate lung injury [60,61]. A lower incidence of VILI has been observed with gradual increases in airway pressure compared to abrupt changes [62]. A recent meta-analysis revealed that employing stepwise increases in PEEP or RMs did not enhance survival or reduce barotrauma [63]. However, stepwise RMs are not recommended [64], as well as abrupt increases in pressures [65,66]. When focusing on PEEP, a recent study showed that lung damage can occur after sustained inflation followed by rapid deflation, which may be caused by hemodynamic impairment and an increase in pulmonary microvascular pressure [67]. The rapid and progressive PEEP release was associated with increased pulmonary arterial pressure and more damage to epithelial cells, regardless of fluid state [68]. In conclusion, it is important to note that slow adjustments to ventilatory parameters are preferable to abrupt changes to minimize further lung damage [69].

### 2.5. Concept of VILI in Clinical Practice

In patients with ARDS, atelectasis is frequent in the dependent areas of the lung, caused by edema in the lung tissue along with the failure of surfactant [70,71,72]. Because there is a smaller volume available for ventilation, for both gas exchange and MV, this condition has led to the abovementioned “baby lung” [73]. The concept of the “baby lung” is a dynamic feature as atelectasis is redistributed toward the ventral regions when patients are placed in the prone position [74,75]. Additionally, even when the lung is aerated, it does not indicate a healthy state; it may still exhibit active inflammation [76]. The key implication is that a reduced V_T_, one which might be considered typical for infants, should be applied to avoid the risk of overinflating the relatively small, normally aerated regions. In fact, the authors of the original ARDS Network trial proposed that lower V_T_ might be necessary to avoid overdistension in ARDS and compared two ventilation strategies. One was a conventional approach, which utilized a V_T_ of 12 milliliters per kilogram (ml/Kg) of predicted body weight (PBW), while the other employed a low-V_T_ strategy, with a V_T_ of just 6 mL/Kg PBW [5]. The results revealed that the low-V_T_ strategy was linked to a significant reduction in mortality, with an absolute decrease in mortality rate of approximately 9 percentage points (approximately 40% in the control group versus 31% in the low-V_T_ group).

In ARDS, regional mechanical variability creates extra stresses that heighten the risk of VILI. The interconnectedness of nearby alveoli means that the collapse or fluid accumulation in one unit causes deformation in adjacent units, as the shared septum is pulled toward the affected area [24,77]. This results in increased shear strain and unequal inflating of neighboring aerated alveoli [78]. In vivo animal studies revealed localized neutrophilic activation in regions of elevated strain [79]. Similar studies in humans have confirmed that lung inflammation in ARDS patients is spatially heterogeneous, a pattern likely due in part to variations in regional mechanical strain [76,80]. A direct link between mechanical inhomogeneity of the lung parenchyma and the increased mortality observed in ARDS patients has been confirmed, reinforcing the critical importance of understanding regional mechanical stresses in disease progression [81].

In this pathophysiological context, both the application of PEEP and the adoption of the prone position can contribute to reducing VILI in patients with ARDS, improving lung homogeneity, and obtaining a more uniform distribution of stress and strain [82,83,84,85,86]. By recruiting collapsed lung areas and preserving alveolar stability, adequate PEEP reduces arterial hypoxemia, avoids collapse or fluid accumulation, and lowers the risk of VILI from atelectrauma [87]. Furthermore, adequate PEEP promotes the redistribution of edematous fluid from the alveoli to the interstitial space, thus reducing intrapulmonary shunt and promoting more uniform interdependent alveolar mechanics [88,89]. However, PEEP also has harmful effects. High levels of PEEP may reduce cardiac output. PEEP, in fact, by increasing pleural pressure, increases the pressure of the right sections of the heart and decreases the pressure gradient for venous return. The reduced venous return lowers the preload of both the right and left ventricles, resulting in a decrease in cardiac output [90,91,92]. Additionally, PEEP may exacerbate lung stress and tension, which may lead to VILI from overdistension. Therefore, lung recruitability is a critical factor in determining the effect of PEEP on an ARDS lung [32,93,94]. Given the role of transpulmonary pressure in VILI, a safe and simple approach would be to use this to set PEEP, through measurement of esophageal pressure as a surrogate for pleural pressure. However, this parameter is measured in less than 1% of ARDS patients [95].

Prone positioning has also been shown to enhance lung homogeneity. In the most dependent parts of the lung, the supine position is linked to the development of gravity-dependent atelectasis in ARDS patients. The increase in lung weight resulting from the accumulation of edema and the compression exerted by the heart and abdominal viscera also determine the compression of the dorsal areas, further contributing to the formation of atelectasis in these lung areas [96]. Because of this, there is a preferential distribution of the VT towards the ventral regions which are therefore at greater risk of overdistension; pleural pressure is in fact higher in the dorsal lung regions than in the ventral ones, so that transpulmonary pressure is higher in the ventral regions [97]. Furthermore, pulmonary perfusion is preferentially distributed towards the dorsal regions, with alteration in the ventilation–perfusion relationship and an increase in the intrapulmonary shunt [96,97,98]. The prone position can attenuate these phenomena, promoting more homogeneous lung ventilation and an improvement in the ventilation–perfusion ratio [74,82,83,99]. Several studies have shown that the prone position improves outcomes of patients with ARDS, especially in patients with greater heterogeneity of lung damage. The PROSEVA multicenter randomized controlled trial demonstrated that placing patients with severe ARDS in the prone position for a minimum of 16 h per day significantly reduced mortality by approximately 17% as compared to semi-recumbent supine position, despite both groups receiving identical lung-protective MV strategies [100]. A more recent meta-analysis of eight randomized clinical trials concluded that prone positioning, when applied for a minimum of 12 h per day, reduced mortality in patients with severe ARDS [101]. The same findings emerged from studies conducted on patients with severe ARDS related to COVID-19 [102,103].

## 3. Clinical Management

The recognition of VILI as a clinical entity that significantly impacts the outcomes of patients with ARDS has significantly changed the philosophy behind mechanical ventilation. Historically, mechanical ventilation aimed to ensure adequate gas exchange and reduce work of breathing. Today, the focus is to achieve adequate gas exchange while minimizing the risk of VILI.

### 3.1. Lung-Protective Ventilation

The advent of lung-protective ventilation strategies, particularly the use of low V_T_ and optimal PEEP, has become a cornerstone of ARDS management. The ARDSNet trial demonstrated that ventilating with low tidal volumes (6 milliliters per kilogram of PBW) significantly reduced mortality compared to conventional V_T_ strategies (12 milliliters per kilogram of PBW) [5]. Starting from 6.5 mL/kg PBW, an increase of 1 mL/kg in V_T_ was associated with a 23% rise in mortality rates among ARDS patients in the ICU [104]. Moreover, patients initially on lower V_T_ (6 mL/kg PBW) had a reduced overall risk of ICU mortality compared to those who started with higher V_T_ (8–10 mL/kg PBW) followed by lower V_T_. The ARDSNet studies, on the other hand, found no link between elevated hospital mortality and ventilation with high V_T_ within the first 48 h [5]. Implementing a lung-protective MV strategy in the emergency department with low V_T_ and P_PLAT_ increased the likelihood of utilizing low V_T_ in the ICU, reducing the risk of VILI and death, according to the LOV-ED investigators [105]. Since higher V_T_ contribute to alveolar overdistention—leading to lung injury and increased mortality—the recommended approach by current guidelines from the European Society of Intensive Care Medicine (ESICM) is to aim for a V_T_ of 4–6 mL/kg PBW, avoiding volumes exceeding 8 mL/kg PBW, while keeping the plateau pressure at or below 30 cmH_2_O [106]. This approach aims to minimize the mechanical forces causing VILI, including overdistension (volutrauma) and barotrauma, while still ensuring adequate ventilation. The clinical application of this strategy has revolutionized ARDS management and drastically improved patient outcomes. Nevertheless, a one-size-fits-all strategy exposes patients to needless injury, which emphasizes the necessity of tailored approaches based on recruitability rather than arbitrary standard thresholds. Indeed, while a low-V_T_ strategy might be ideal for some patients, it could be dangerous for others. The reliance on PBW is founded on the questionable assumption that it accurately reflects lung size. However, critically ill patients, particularly those with ARDS, often do not present normal lung volumes. In this scenario, it may be more suitable to adjust V_T_ based on compliance of the respiratory system, which more accurately resembles the functional lung capacity available for ventilation [107].

Despite the widespread adoption of low-V_T_ ventilation since its inception in 2000, the mortality rate associated with ARDS has not shown a significant decline. In certain instances, mortality rates may even parallel those observed in patients who are ventilated with higher V_T_. This raises important questions about the efficacy of current ventilation strategies and highlights the need for further research to optimize ARDS management and improve patient outcomes [95]. Secondary analyses of ARDS Network data further revealed that non-eligible patients ventilated at approximately 10 mL/kg PBW exhibited mortality rates comparable to those receiving low V_T_, and that patients with preserved respiratory system compliance paradoxically experienced increased mortality with low-V_T_ ventilation. In this subgroup, higher V_T_ was associated with improved outcomes [108]. This finding introduces a provocative paradigm: the risk-benefit balance of tidal volume may depend not solely on body weight-derived estimates, but rather on the underlying mechanical properties of the lung. Stratifying patients based on compliance, rather than adopting a uniform low-V_T_ approach, may thus be a more physiologically rational and potentially outcome-improving strategy. These data challenge the prevailing assumption that low V_T_ is universally protective and underscore the necessity for a compliance-guided, individualized approach.

In parallel, the theoretical foundation of open lung strategies—combining low V_T_ with RMs and elevated PEEP—remains compelling, particularly when considering that the principal mechanisms of VILI include repetitive alveolar recruitment and derecruitment and regional overdistension due to stress multipliers at heterogeneously aerated interfaces. Nevertheless, the ART trial reported increased mortality in patients treated with an open lung protocol, likely attributable to the transient and incomplete nature of the lung recruitment achieved. Subsequent analyses demonstrated a failure to sustain lung opening, as evidenced by declining respiratory compliance and gas exchange parameters within one-hour post-intervention [65].

High-Frequency Oscillatory Ventilation (HFOV) was long considered a potential means to achieve an “open lung” condition while minimizing tidal strain by delivering very small tidal volumes at supraphysiological frequencies. However, two major randomized controlled trials—OSCILLATE and OSCAR—challenged this hypothesis and led to a broad abandonment of HFOV in ARDS management [109,110]. The OSCILLATE trial compared early HFOV with conventional lung-protective ventilation in moderate to severe ARDS. The trial was stopped early for harm, as the HFOV group had significantly higher mortality (47% vs. 35%) and more patients in the HFOV group received vasoactive drugs and received them for a longer period than did patients in the control group, suggesting hemodynamic compromise induced by high mean airway pressures [109].

In contrast, the OSCAR trial, compared HFOV not to a standardized lung-protective protocol but rather to usual care in the participating ICUs. The OSCAR trial found no significant difference in 30-day mortality between groups, but it did not identify any benefit of HFOV either [110].

Taken together, these trials suggest that while HFOV can maintain alveolar recruitment through high mean airway pressures, it may do so at the cost of adverse hemodynamic effects, and crucially, neither trial demonstrated a survival benefit. Importantly, neither OSCILLATE nor OSCAR showed conclusive evidence that HFOV successfully achieved a fully and stably recruited lung. This raises the possibility that the failure of HFOV lies not in the physiological rationale of open lung ventilation, but in the inability of the applied protocols to realize the recruitment necessary to gain its protective benefits. Consequently, these findings cast doubt not on the concept of open lung protection per se, but on the methods used to operationalize it in clinical trials.

Given the viscoelastic nature of alveolar tissue, ventilation modes that modulate inspiratory and expiratory time may better support alveolar stabilization. Airway Pressure Release Ventilation (APRV), which utilizes prolonged inspiratory phases and brief releases, has shown promise in promoting lung protection by maintaining alveolar openness. A recent meta-analysis indicates that APRV may offer substantial lung protection, with improved oxygenation and decreased incidence of VILI compared to conventional modes [111]. These findings suggest that APRV, by optimizing inspiratory and expiratory time ratios, may represent a promising alternative in the context of personalized lung-protective ventilation. Nevertheless, high-quality, adequately powered RCTs are required to establish their definitive role in ARDS management.

#### 3.1.1. Positive End-Expiratory Pressure

The optimization of PEEP settings remains a critical yet challenging aspect of lung-protective ventilation in ARDS. PEEP plays a pivotal role in maintaining alveolar recruitment and minimizing atelectrauma; however, excessive PEEP may cause overdistension of aerated lung regions and hemodynamic compromise, highlighting the need for precise titration to balance benefits and risks. Four major randomized clinical trials (ART [65], ALVEOLI [112], ExPress [113], and LOV trials [34]) encompassing a total of 3264 patients, have evaluated the effects of higher PEEP, around 15 cm H_2_O, compared to lower PEEP levels (approximately 8 cm H_2_O, or 13 cm H_2_O in the ART trial). None of these trials showed a survival benefit with higher PEEP, even though one suggested a potential advantage for sicker patients [114]. Notably, the ART trial indicated an increase in mortality when higher PEEP levels were used. This was not only a result of high PEEP; an intensive RMs strategy was also included in the protocol. These conflicting findings may be attributed to several factors. Differences in trial design, patient selection criteria, and the heterogeneous nature of ARDS populations can impact outcomes and the generalizability of results. For example, applying uniform PEEP levels without accounting for individual lung recruitability can result in overdistension and subsequent lung injury [94]. Moreover, high PEEP may negatively affect hemodynamics by increasing shunt, dead space, and right ventricular afterload, while reducing cardiac output [115,116].

Because of its technical difficulty and lack of broad training, esophageal pressure is rarely used in clinical practice as a surrogate for pleural pressure, despite it may provide a more customized method of titrating PEEP [117].

The direct correlation between lung recruitability and CT-derived PEEP levels is not so strong, although useful to assess alveolar recruitment and de-recruitment [118]. Despite being the gold standard method for assessing recruitability, CT scans lack repeatability and availability at the bedside.

With a re-aeration score that correlates with better oxygenation and recruitment, lung ultrasonography has emerged as a valuable method for evaluating alveolar recruitment [119,120]. It is unable to assess lung hyperinflation, though. Through the identification of opening and closing pressures in atelectatic zones, lung ultrasound may help assess response to RMs. Running an incremental and decremental PEEP trial, the opening and closing pressures can be easily identified as the points where the consolidation pattern disappears and re-appears. To improve alveolar recruitment, PEEP should be adjusted around 2 cm H_2_O above the closing pressure [121].

Electrical impedance tomography (EIT) is a noninvasive bedside tool that provides real-time insights into lung impedance, indicating lung volume changes [122,123]. EIT allows for the detection and quantification of regional alveolar overdistention and collapse, aiding in individualized PEEP titration [124,125].

In a recent RCT, EIT-guided PEEP titration led to a disconnect between PEEP and FiO_2_ settings, but did not result in significant long-term clinical outcome differences compared to conventional PEEP/FiO_2_ tables [126]. Another trial showed that EIT-guided PEEP titration resulted in lower PEEP levels and improved survival rates compared to settings based on pressure-volume loops [127]. Additionally, a recent randomized crossover study indicated that EIT-guided PEEP titration significantly reduced mean pulmonary artery pressure compared to the High-PEEP/FiO_2_ strategy in patients with moderate-to-severe ARDS [128]. Nevertheless, the widespread implementation of EIT in ARDS management is hindered by the elevated cost of the device, the requirement for advanced operator training for signal acquisition and interpretation, and the critical importance of precise electrode belt placement to ensure data accuracy.

In the LIVE trial, PEEP levels and ventilation strategies were individualized based on lung morphology. The control group received a V_T_ of 6 mL/kg PBW and PEEP per a low PEEP-FiO_2_ table, while the customized group with focal ARDS had a V_T_ of 8 mL/kg, minimal PEEP, and prone positioning tailored to imaging assessments. For non-focal ARDS, high PEEP and a V_T_ of 6 mL/kg were used. The study found no mortality difference in moderate-to-severe ARDS patients between personalized and traditional low-V_T_ ventilation. Misclassification of lung morphology occurred in 21% of patients, leading to higher mortality rates. However, a per-protocol analysis of accurately classified patients showed a significant survival benefit for the personalized ventilation group [129]. Future trials should focus on improving the accuracy of lung morphology classification to enhance personalized ventilation strategies and explore their impact on patient outcomes in ARDS.

#### 3.1.2. Prone Position

A promising development in ARDS management was prone positioning. Several randomized controlled trials have confirmed the efficacy of prone positioning in improving oxygenation and survival in patients with severe ARDS [100,101]. The physiological rationale of the prone position and its impact on the outcomes of ARDS patients have already been discussed in Section 2.5. “Concept of VILI in clinical practice”.

#### 3.1.3. Ultra–Lung-Protective Ventilation and Extracorporeal Life Support (ECLS)

The ARDS Network trial found that restricting V_T_ to 6 mL/kg PBW and maintaining P_PLAT_ ≤ 30 cmH_2_O improves survival but may not fully protect all patients’ lungs [5,35,130]. Reducing V_T_ further to below 4 mL/kg PBW and P_PLAT_ below 25 cmH_2_O, known as “ultra-lung-protective ventilation”, may decrease VILI and improve outcomes, although it carries risks of severe respiratory acidosis and complications such as increased intracranial pressure and reduced organ perfusion [131,132,133].

Extracorporeal life support (ECLS), including ECMO and extracorporeal carbon dioxide removal (ECCO_2_R), can manage respiratory acidosis. ECMO is more invasive, while ECCO_2_R specifically targets carbon dioxide removal without significant oxygenation [134,135]. Clinical studies have shown both techniques can facilitate ultra-lung-protective ventilation. For instance, the SUPERNOVA pilot trial demonstrated that ECCO_2_R allowed a V_T_ of 4 mL/kg PBW and P_PLAT_ < 25 cmH_2_O while maintaining acceptable pH and arterial partial pressure of carbon dioxide (PaCO_2_), thereby reducing airway driving pressure and respiratory rate, potentially lowering VILI risk [136].

The EOLIA trial compared ECMO to optimal conventional management in severe ARDS patients, suggesting a trend toward reduction in mortality with ECMO despite not being significant [137]. However, the trial’s unblinded design, high crossover rate, and early termination due to futility limit the interpretation of these findings [138]. The ESICM currently recommends against using ECCO_2_R outside of randomized clinical trials [106].

#### 3.1.4. Management of Sedation and Neuromuscular Blocking Agents and Early Transitioning from Controlled to Assisted Ventilation

Patients with ARDS often experience extreme dyspnea and may “fight the ventilator”, aggravating VILI. Administering a neuromuscular blocking agent ensures patient-ventilator synchrony, facilitating the limitation of V_T_ and pressure. A multicenter, placebo-controlled, randomized trial with 340 ARDS patients found that those treated with a neuromuscular blocking agent for 48 h had reduced 90-day mortality compared to the placebo group, without significant residual muscle weakness. The difference in mortality became apparent approximately 16 days after treatment began, likely due to improved patient-ventilator synchrony, which may decrease mechanical lung stress and the associated risks of biotrauma and multiorgan failure [139,140]. Furthermore, a previous study showed that early use of neuromuscular blockade decreases the proinflammatory response in patients with ARDS ventilated with a lung-protective strategy [141]. A recent meta-analysis of five randomized controlled trials with 1461 patients found that neuromuscular blockade in moderate to severe ARDS improved oxygenation only after 48 h and reduced the risk of VILI without increasing ICU-acquired weakness. However, it did not result in more ventilator-free days, shorter overall duration of mechanical ventilation, or reduced mortality, regardless of ARDS severity [142].

To mitigate the effects of deep sedation and neuromuscular blockade used for achieving patient-ventilator synchrony, assisted ventilation modalities are increasingly employed during the early phase of ARDS management. Transitioning from controlled to assisted ventilation is complex and influenced by factors such as ICU-acquired weakness, ventilator-induced diaphragmatic dysfunction, and PSILI [143,144,145]. PSILI, in particular, is an important complication in ARDS patients who exert spontaneous breathing efforts during mechanical ventilation. It occurs when strong inspiratory efforts lead to significant oscillations in transpulmonary pressure, increasing the risk of regional volutrauma and atelectrauma. Additionally, these efforts can create exaggerated negative pleural pressures, promoting pulmonary edema through increased transvascular fluid filtration due to a heightened hydrostatic-pleural pressure gradient [145]. These mechanisms highlight the importance of effectively managing the transition to assisted ventilation [146]. A successful transition to assisted ventilation can reinstate spontaneous respiratory activity, improve oxygenation, mitigate diaphragmatic atrophy, and lower the risks associated with prolonged controlled mechanical ventilation and deep sedation [147,148,149,150,151,152]. Nonetheless, this transition remains challenging due to the complex pathophysiology of ARDS, which predisposes patients to respiratory muscle fatigue and distress [153]. To optimize this transition, careful attention must be given to factors such as respiratory effort, sedation levels, the PaO_2_/FiO_2_ ratio, and the selection of the most suitable ventilation mode [154]. Different modes of assisted ventilation have been found to enhance lung function and decrease inflammation, apoptosis, and fibrogenesis, as well as less epithelial and endothelial cell damage when compared to pressure-controlled ventilation [155,156,157,158].

#### 3.1.5. ARDS Biological Subphenotypes and Pharmacologic Interventions

ARDS is characterized by different pathogenic events resulting in similar clinical presentations. These clinical manifestations, although similar, are thought to represent distinct phenotypes and genotypes, laying the foundation for a personalized therapeutic approach to the individual patient. There are now two recognized ARDS phenotypes: hyperinflammatory and hypoinflammatory. Each has unique clinical outcomes and therapeutic responses. These phenotypes were reliably distinguished by post hoc latent class analysis of five RCTs using clinical characteristics and blood biomarkers associated with coagulopathy, endothelial damage, and inflammation [159,160,161,162,163]. The same phenotypic difference was also supported by observational research that used unsupervised cluster analysis based only on biomarker profiles, without clinical variables [164]. Notably, these phenotypes showed different and sometimes even opposite responses to PEEP, fluid management, and pharmacologic treatment [159,160,161,162,163].

Research on mesenchymal stem cells and anti-inflammatory therapies has mostly focused on animals [165,166,167]. These treatments have the potential to lower the incidence of VILI since they can target inflammatory processes before mechanical ventilation is started. By attenuating the inflammatory cascade, these treatments could prevent or reduce the systemic inflammatory response, thereby limiting the progression of VILI and protecting against multiorgan dysfunction. Additionally, mesenchymal stem cells have been explored for their potential to regenerate lung tissue and reduce fibrosis following lung injury. Two recent studies in humans showed that the use of mesenchymal stem cells was safe in patients with moderate to severe ARDS [168,169]. However, clinical utility in humans is yet to be proven and larger trials are needed to assess efficacy.

#### 3.1.6. Emerging Insights in Biological Subphenotyping for ARDS Management

Recent advances in understanding the mechanisms behind VILI have opened new potential approaches to improve patient outcomes. Implementing biological subphenotypes into clinical practice is empowering ARDS management [170].

For instance, an experimental study demonstrated that aerobic exercise, known for its benefits in chronic respiratory diseases, can reduce VILI by inhibiting the activation of Protein kinase C alpha (PKCα) and NOD-like receptor protein 3 (NLRP3) [171,172]. This suggests that aerobic exercise may serve as a preventive measure for ARDS patients, although further studies are needed to provide clinical evidence. Emerging research also highlights the dysregulation of exosomal microRNAs (miRNAs) as a significant factor in the pathophysiology of respiratory diseases, including ARDS [173,174].

Limited studies have examined circulating miRNAs in this context. One study found elevated levels of circulating miR-122 in ARDS patients compared to controls, linking this increase with 30-day mortality and acute liver injury [175]. Another study indicated higher serum miR-92a expression in sepsis-related ARDS patients than in those with sepsis alone [176]. Additionally, three dysregulated miRNAs—miR-424, miR-92a, and miR-181a were identified in blood samples, with inflammatory markers miR-181a and miR-92a upregulated in ARDS patients, while the anti-inflammatory marker miR-424 was downregulated, suggesting a protective role [177]. Furthermore, a study on ten miRNAs associated with ARDS found that four—miR-146a, miR-27a, miR-126, and miR-155—were differentially expressed in ARDS patients compared to non-ARDS controls. This emerging evidence positions miRNAs as promising diagnostic biomarkers and potential therapeutic targets for modulating inflammatory and repair processes to mitigate VILI.

#### 3.1.7. Critical Perspectives and Future Directions

Despite significant advances in understanding and managing VILI in ARDS, several critical challenges and uncertainties remain. While lung-protective ventilation strategies such as low V_T_ of 6 mL/kg PBW and higher PEEP with rescue RMs have become standard practice, clinical trials have shown inconsistent survival benefits, particularly regarding PEEP titration. Recent efforts have introduced targets like lower ΔP and reduced MP to minimize VILI [5,34,65,106,112,113]. However, the blanket application of low V_T_ may not be suitable for all patients, and individualization of PEEP is necessary, as high PEEP can be harmful to non-recruitable lung tissue. A one-size-fits-all approach fails to account for the complexities of ARDS patients. A refined understanding is needed to individualize ventilation strategies, emphasizing the importance of individual physiological characteristics over standardized protocols.

Moreover, while higher PEEP has theoretical benefits, clinical trials have yielded mixed results. It may be more effective to tailor PEEP based on lung recruitability rather than fixed thresholds. Emerging studies are exploring individualized PEEP strategies, highlighting the need for a selective approach rather than a routine application [178].

Additionally, past research indicates that ventilation strategies combining low V_T_, high PEEP, and permissive hypercapnia can significantly reduce mortality in ARDS patients [179,180]. However, recent evidence suggests that respiratory rate management is essential to minimize MP and ensure effective ventilation, thus contrasting with the concept of permissive hypercapnia which is based on the application of low tidal volume with high respiratory rates [53,54,55,56,57].

Future research should prioritize personalized ventilation strategies integrating multimodal monitoring—including esophageal pressure, MP, EIT, and lung ultrasound—to tailor interventions more precisely to lung recruitability and mechanics. Ongoing trials will certainly clarify possible weaknesses of current lung-protective MV strategies, like PERMISS aim to investigate the effects of permissive ventilation compared to conventional methods on ventilation-free days, while the ACTiVE trial will soon reveal results on automated ventilation’s impact in the ICU [181].

The recent identification of distinct biological subphenotypes in ARDS highlights the need to transition from traditional clinical classifications to a precision medicine approach [159,160,161,162,163]. The varying responses of hyperinflammatory and hypoinflammatory subphenotypes to different ventilation settings and pharmacological treatments suggest that incorporating biomarker profiling into routine care could enhance therapy and improve outcomes. To this end, large-scale randomized controlled trials (RCTs) are essential for validating subtype-guided treatment algorithms and exploring novel therapies, such as mesenchymal stem cells and targeted anti-inflammatory agents.

Emerging technologies like artificial intelligence (AI) offer promising opportunities to enhance patient-specific decision-making in critical care [182]. AI algorithms can analyze complex clinical and physiological data in real time to optimize ventilator settings and reduce the risk of VILI [183,184,185]. However, despite preliminary successes, the use of AI in this context remains largely in the exploratory phase, necessitating robust prospective studies and clinical trials to validate these tools and ensure their efficacy and safety for widespread clinical use.

Additionally, new mechanistic insights, such as the protective effects of aerobic exercise and the regulatory role of exosomal microRNAs, open intriguing possibilities for adjunctive therapies aimed at modulating inflammatory pathways and promoting tissue repair [171,172,173,174,175,176,177]. These innovative approaches, though still in the early stages, warrant further exploration through well-designed clinical trials.

In summary, future directions in ARDS management lie in integrating advanced monitoring, biological subphenotyping, and computational tools to refine personalized ventilation strategies. This multifaceted approach holds the promise of not only reducing VILI but also improving overall survival and long-term outcomes for this challenging patient population.

## 4. Conclusions

The multifactorial nature of VILI—encompassing barotrauma, volutrauma, atelec-trauma, biotrauma, and, more recently, ergotrauma and injury driven by heterogeneous lung mechanics—underscores the complexity of ventilating an already injured lung. The identification of these mechanisms has led to the implementation of lung-protective strategies such as low-VT ventilation, individualized PEEP titration, and prone positioning, which have markedly improved clinical outcomes. Despite these advances, no single approach universally prevents VILI, and the optimal mechanical ventilation strategy remains elusive. Advances in personalized ventilatory management, guided by patient-specific mechanics and real-time monitoring (e.g., transpulmonary pressure, MP, and imaging techniques), open the door to truly personalized ventilation strategies that may further mitigate lung injury. Moreover, novel approaches such as ultra–lung-protective ventilation enabled by extracorporeal support, and future developments in artificial intelligence and biomarker-driven phenotyping, offer exciting opportunities to refine therapy and improve survival. Moving forward, a paradigm shift is needed: from a one-size-fits-all approach to precision medicine in ARDS. Until then, clinicians must continue to rely on established lung-protective strategies, while remaining attuned to the evolving evidence that may guide the future of mechanical ventilation.

## Data Availability

No new data were created or analyzed in this study.

## Abbreviations

ARDS	Acute Respiratory Distress Syndrome
CI	Confidence Interval
CT	Computed Tomography
ECLS	Extracorporeal Life Support
ECCO_2_R	Extracorporeal Carbon Dioxide Removal
ECMO	Extracorporeal Membrane Oxygenation
EELV	End-Expiratory Lung Volume
EIT	Electrical Impedance Tomography
FiO_2_	fraction of inspired oxygen
HBO	Hyperbaric Oxygen
HCT	Hematocrit
ICU	Intensive Care Unit
IL	Interleukin
LPV	Lung-Protective Ventilation
MP	Mechanical Power
MV	Mechanical Ventilation
PaCO_2_	Partial Pressure of Carbon Dioxide
PACO_2_	Alveolar Partial Pressure of Carbon Dioxide
PEEP	Positive End-Expiratory Pressure
PET	Positron Emission Tomography
POCUS	Point-of-Care Ultrasound
PROSEVA	Proning Severe ARDS Patients
RCT	Randomized Controlled Trial
RM	Recruitment Maneuver
RR	Respiratory Rate
SUPERNOVA	Study on Ultra-Protective Ventilation and Extracorporeal Life Support
TGF	Transforming Growth Factor
TNF	tumor necrosis factor
Transp	Transpulmonary Pressure
V/Q	Ventilation/Perfusion Ratio
VILI	Ventilator-Induced Lung Injury
V_T_	Tidal Volume
∆P	driving pressure
